# The Effect of Hatchery Release Strategy on Marine Migratory Behaviour and Apparent Survival of Seymour River Steelhead Smolts (*Oncorhynchus mykiss)*

**DOI:** 10.1371/journal.pone.0014779

**Published:** 2011-03-29

**Authors:** Shannon Balfry, David W. Welch, Jody Atkinson, Al Lill, Stephen Vincent

**Affiliations:** 1 Faculty of Land and Food Systems, The University of British Columbia, Vancouver, British Columbia, Canada; 2 Kintama Research Services Ltd., Nanaimo, British Columbia, Canada; 3 Department of Zoology, The University of British Columbia, Vancouver, British Columbia, Canada; 4 Living Rivers–Georgia Basin/Vancouver Island, North Vancouver, British Columbia, Canada; 5 Seymour Salmonid Society, North Vancouver, British Columbia, Canada; National Oceanic and Atmospheric Administration/National Marine Fisheries Service/Southwest Fisheries Science Center, United States of America

## Abstract

Early marine migratory behaviour and apparent survival of hatchery-reared Seymour River steelhead (*Oncorhynchus mykiss*) smolts was examined over a four year period (2006–2009) to assess the impact of various management strategies on improving early marine survival. Acoustically tagged smolts were released to measure their survival using estuary and coastal marine receivers forming components of the Pacific Ocean Shelf Tracking (POST) array. Early marine survival was statistically indistinguishable between releases of summer run and winter run steelhead races, night and day releases, and groups released 10 days apart. In 2009, the survival of summer run steelhead released into the river was again trialed against groups released directly into the ocean at a distance from the river mouth. Apparent survival was improved significantly for the ocean released groups. The health and physiological status of the various release groups were monitored in years 2007–2009, and results indicate that the fish were in good health, with no clinical signs of disease at the time of release. The possibility of a disease event contributing to early marine mortality was further examined in 2009 by vaccinating half of the released fish against common fish diseases (vibriosis, furunculosis). The results suggest that marine survival may be enhanced using this approach, although not to the extent observed when the smolts were transported away from the river mouth before release. In summary, direct experimental testing of different release strategies using the POST array to measure ocean survival accelerated the scientific process by allowing rapid collection of data which enabled the rejection of several existing theories and allowed tentative identification of several new alternative approaches that might improve early marine survival of Seymour River steelhead.

## Introduction

In recent years, steelhead populations in the Seymour River as elsewhere in the Strait of Georgia and the eastern side of Vancouver Island have declined substantially [1]. This decline has been best documented on the Keogh River (NE Vancouver Island), where ocean survival (which averaged 15% in the mid 1980's) has now declined to levels well below the 4% generally regarded as being necessary to maintain a stable population [2]. While ocean regime shifts driven by atmospheric factors such as the Pacific decadal oscillation undoubtedly play a role in this decline [3], [4], direct comparison of steelhead survival from otherwise apparently similar rivers flowing east into the “Salish Sea” ecosystem (Puget Sound, Strait of Georgia, Johnstone and Queen Charlotte Straits) and west into the west coast shelf ecosystem where adult return rates were much higher, indicates that other factors play important roles [5].

**Figure 1:**
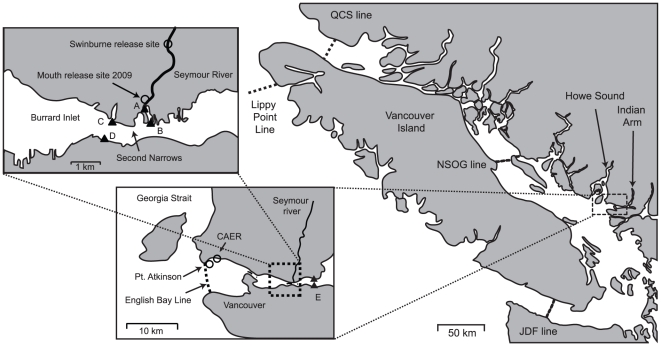
Maps of the study area, illustrating release sites and receiver locations using three separate scales. In these three maps, release sites are shown as circles, listening lines as dotted lines, and locally-deployed receivers as filled triangles. On the right is the Strait of Georgia study area at the greatest scale which shows the positions of the POST listening lines relevant to this study: the NSOG (Northern Strait of Georgia), the QCS (Queen Charlotte Strait), the JDF (Strait of Juan de Fuca), and the Lippy point line. The centre figure shows the Seymour River and the receiving waters of Burrard Inlet. The two circles represent the seawater (SW) release sites: Point Atkinson (2008, 2009) and CAER (2009). The two triangles show the locations of the two VR2's deployed in 2007 to monitor movement into Indian Arm. The dotted line represents the position of the 13-element English Bay line of VR2's deployed in 2007 to monitor egress from Burrard Inlet. The leftmost figure shows the lower portion of the Seymour River from the river release site at Swinburne to the mouth 2.7 km downstream. The locations of local VR2's to monitor the tagged fish in the immediate marine environment are shown as triangles with a letter beside them. A (estuary) was present during all releases from 2006 to 2009; B (outer) was present in 2008 and 2009; C (North Shore Burrard Inlet) and D (South Shore Burrard Inlet) were present in 2006 and 2009. E (Indian Arm) were present in 2007 only.

Before the advent of acoustic tags and the associated receiver technology, there was very little ability to monitor specific salmon stocks in the marine environment and no practical ability to measure their survival on time and spatial scales exceeding that of the river phase of the migration. However, with the deployment of large-scale networks such as the Pacific Ocean Shelf Tracking (POST) array it is now possible to track individual fish and salmon stocks over hundreds and potentially thousands of kilometers [6], [7], [8], [9], [10]. The Seymour River, which is located in North Vancouver, British Columbia, Canada, is geographically well-placed for studies utilizing the POST array. The migration and survival of Seymour steelhead (*Oncoryhynchus mykiss*) smolts leaving the river can be tracked for at least the first 150 km of their southern migration through Juan de Fuca Strait (JDF) or over a distance of 400 km if the smolts migrate north and exit via the Queen Charlotte Strait (QCS) (Fig. 1). By comparing the number of fish released, with the number subsequently detected in Burrard Inlet, the Northern Strait of Georgia (NSOG), QCS and JDF we can compare how apparent survival varies as a function of time and distance for different groups of fish released from the Seymour River.

The overall survival of hatchery-released Seymour steelhead has recently averaged about 0.5%, with only 100 fish returning out of the 20,000 typically released (Seymour Salmonid Society, unpublished data). This study was initiated to understand the pattern of migration, the rate of mortality experienced along that migration pathway, and possible reasons for the observed mortality. Numerous experimental groups of fish were released from 2006–2009 to test the effect of various release strategies on migratory behaviour and survival. The objective of this multi-year progressive study was to compare the migration and survival of different experimental groups within each year, and develop novel release strategies aimed at improving marine survival and adult return rates to the hatchery. In an attempt to determine the possible cause(s) of marine mortality, the various experimental release groups in 2007–2009 were sampled to measure fish health using numerous physiological parameters, including tissue samples analyzed for pathogen prevalence.

## Materials and Methods

### Ethics Statement

All work involving live fish reported in this paper was annually reviewed and pre-approved by institutional Animal Care Review Committees as meeting or exceeding the standards laid out by the Canadian Council on Animal Care. Over the course of this four year project, protocols were approved by the Pacific Region Animal Care Committee of Fisheries and Oceans Canada, the Animal Care Committee of Vancouver Island University, or by the University of British Columbia Animal Care Committee.

### Study Location

The Seymour River in North Vancouver, British Columbia, Canada, is a medium-sized stream draining an area of approximately 176 km^2^. Despite its proximity to a major city, the river retains a fairly wild character apart from the industrial development at the mouth. Maps of the study area using three separate scales are presented in Figure 1.

In 1981, the Seymour Hatchery was built just below the Seymour River dam and has been used to augment populations of steelhead for terminal recreational harvest opportunities [11] and other resident salmonids. The steelhead program has remained fairly constant since the inception of hatchery operations with a total of approximately 20,000 smolts being reared in each brood cycle, with the majority being summer run fish. Both summer run and winter run races are present in the Seymour River. Summer run adults return in the summer to early fall, and hold over in freshwater until spawning. In contrast, the winter run adults only return in the winter. Wild broodstock (distinguished from hatchery fish by the presence of an intact adipose fin) are caught by anglers on rod and line and transported to the hatchery where they are spawned (typically summer runs in March and winter runs in April). Fry are adipose-clipped and reared in outside natural channels until their release as one year old fish. At release time, the smolts are transported by truck for release into the lower river (conventional release site called Swinburne) located 2.7 km from the river mouth (approximately 20 min drive). Transporting the fish from the hatchery was implemented to avoid both inter-species and intra-species competition in the river and reduce the possibility of residualisation. Transports were performed by netting the fish out of their rearing channel and into a specialized fish transport tank truck, containing aerated hatchery water. The density during each transport was approximately 41–45 kg/m^3^ (maximum capacity of the truck). Once at the release site, one end of a large hose (∼20 cm diameter, 18 m long) was attached to the tank outlet and the other end placed into the river. A valve was released and the fish quickly flushed out of the tank through the hose and into the river, a process taking just a few minutes.

### Tagging

Steelhead smolts were surgically implanted with V9-6L acoustic transmitters (9×21 mm, mass in water 1.6 g, frequency 69 kHz, 30–90 s random delay, Vemco Ltd., Halifax, Canada), except in the 2006 study where high acoustic power, V9-1H acoustic transmitters (9×24 mm, mass in water 2.2 g, frequency 69 kHz, 30–90 s random delay, Vemco Ltd., Halifax, Canada) were used. The rated battery life of the transmitters (or “tags”) as specified by the manufacturer, varied in each year but was typically between 3∼4 months of life for the V9-6L tags. However our results from annual testing of a sub-sample of tags, indicated that mean times to failure of 8∼9 months for the V9-6L tags. All surgeries were performed at the Seymour hatchery using previously described protocols [8], [12]. Briefly, fish were anaesthetized in buffered tricaine methane sulphonate (TMS, Syndel Laboratories, Vancouver, Canada; 70 ppm TMS; 140 ppm NaHCO_3_) and placed ventral side up on a surgery board. The gills were continuously irrigated with a gentle flow of water containing a maintenance dose of anaesthetic (50 ppm TMS, 100 ppm NaHCO_3_) throughout the procedure. Tags were inserted through a midventral incision and closed with two or three polydioxanone monofilament sutures. Tagged fish receiving different treatments were held together in separate release tanks after the surgeries for approximately one to two weeks prior to release. The fish were monitored during this recovery period for mortalities (no post-implantation mortalities observed), tag shedding or abnormal swimming behaviour. At all times, tag:body size ratios were below the recommended limits of 16% of length and 8% of body weight [13].

### Acoustic Telemetry

The marine migratory behaviour of the tagged fish was monitored using the POST array (primarily the NSOG and QCS line) and temporary VR2 receiver lines located in English Bay, Indian Arm, the river mouth and estuary (the location of these varied with year, details provided below). A description of the POST marine telemetry array and detection efficiencies have been described elsewhere [7], [8], [14], [15], [16], [17], [18]. The overall detection efficiency of the innermost river mouth receiver appeared to approach 100%, as there were no detections of river-released fish on the QCS or NSOG receiver lines that had not previously been seen on this receiver. There were however 3 fish in 2009 that were detected on the outer river receiver but not the inner. The detection efficiency of the V9-6L acoustic tags crossing the main coastal ocean sub-arrays employed in this study was 80∼90% [16].

### Release Strategies

This project was started in 2006 to (initially) examine and compare steelhead migration and survival in groups of fish released in the dark versus daylight. The poor survival of steelhead prompted the need for additional studies to further examine different release strategies. The results from each year were used to develop and implement new strategies designed to improve the survival of released steelhead. The use of the POST array enabled the timely collection of migration survival data that could be employed to modify the release strategies for testing the following year. This project therefore involved a progression of yearly studies, each building upon the new knowledge gained from the results of the previous year's study. The details of the 2006–2009 releases and sampling procedures performed in each year are presented separately below. An overview of the different release groups, release dates and locations, and other tagging information is presented in Table 1.

**Table 1:** Description of the various release strategies, strains and experimental treatment groups of tagged Seymour steelhead smolts examined from 2006–2009.

Year	Release Strategy	Treatment/Strain	No. Tagged	Release Date/Time (PST)	Fork Length ± sem (mm)
2006	FW: River-Night	Summer run	13	May 10/2045 h	217.6±3.07
	FW: River-Night	Winter run	12	May 10/2045 h	196.3±2.30
	FW: River-Day	Summer run	13	May 10/1400 h	221.9±3.01
	FW: River-Day	Winter run	12	May 10/1400 h	191.7±2.31
2007	FW: River-Early	Summer run	10	April 27/1415 h	187.4±3.91
	FW: River-Early	Winter run	10	April 27/1415 h	188.5±3.91
	FW: River-Normal	Summer run	10	May 8/1350 h	190.8±5.14
	FW: River-Normal	Winter run	10	May 8/1350 h	181.0±3.07
	FW: River-Late	Summer run	10	May 15/1100 h	183.0±3.86
	FW: River-Late	Winter run	10	May 15/1100 h	184.4±3.91
2008	FW: River	Summer run	15	May 13/1415 h	174.8±2.70
	FW: River	Winter run	15	May 13/1415 h	193.5±2.57
	SW: Barged	Summer run	15	May 16/1500 h	174.5±2.51
	SW: Barged	Winter run	15	May 16/1500 h	191.7±3.57
2009	FW: River	Unvaccinated	19	May 21/1130 h	165.7±2.92
	FW: River	Vaccinated	18	May 21/1130 h	169.7±1.94
	FW: Mouth	Unvaccinated	19	May 14/1448 h	162.9±2.09
	FW: Mouth	Vaccinated	18	May 14/1448 h	170.3±2.44
	SW: Barged	Unvaccinated	19	May 20/1600 h	172.4±2.92
	SW: Barged	Vaccinated	19	May 20/1600 h	171.0±1.92
	SW: Marine	Unvaccinated	19	May 20/1444 h	168.4±2.00
	SW: Marine	Vaccinated	19	May 20/1444 h	172.7±2.01

The 2006 steelhead were implanted with V9-1H acoustic tags and the 2007–2009 fish were implanted with V9-6L acoustic tags.

#### 2006 Study

A total of 50 tagged steelhead (equal numbers of summer and winter run) were released on May 10^th^ along with approximately 5,000 untagged fish (summer run 204.7±18.0 mm, winter run 183.7±11.0 mm) at the conventional release point in the river (Swinburne). To examine the role of release time on fish survival, half of the fish were released in the daylight (1400 hr PST local time) and the other half released later that day in the dark (2045 hr PST local time). A single VR2 receiver placed at the mouth of the river, monitored entry of the fish into the marine environment. The permanent array of POST listening lines in JDF, NSOG, and QCS were used to determine the marine migratory behaviour and survival. These fish were not sampled to determine health or physiological status.

#### 2007 Study

In 2007, a total of 60 hatchery steelhead (equal numbers of summer and winter run) were tagged and released along with approximately 5,000 untagged fish (summer run 190.3±12.3 mm, winter run 196.8±17.3 mm) in the river (at Swinburne as described above). To examine the effect of release timing, one-third of the fish were released on each of 3 different dates approximately 10 days apart (April 27, May 8, May 15; 10 each of summer and winter run on each date). As in 2006, a single VR2 receiver located at the river mouth monitored entry into the marine environment. To better assess marine migratory behavior, two VR2's were placed east of the river mouth to monitor entry into Indian Arm, a large blind fjord east of Burrard Inlet. To monitor the entry of fish into Georgia Strait, a total of thirteen receivers were placed in a line across English Bay, approximately 20 km west of the Seymour River. The permanent array of POST listening lines was used to determine the larger scale migration pathway and survival.

Health assessments were performed on the fish sampled at the hatchery, approximately 2–5 weeks prior to their release. A total of 60 untagged fish (equal numbers of summer and winter) were randomly sampled without replacement from their rearing tanks. The methodologies used are described in detail below. Briefly, each fish was euthanised, measured, bled, and necropsied. Tissue samples were taken to assess smoltification status and pathogen prevalence.

#### 2008 Study

A total of 60 steelhead were tagged (equal numbers of summer and winter run). At the time of release, 30 tagged smolts were released (as previously described) into the river (at Swinburne), along with approximately 5,000 untagged fish (summer run 173.8±15.3 mm, winter run 187.5±30.7 mm). The remaining 30 tagged fish (15 equal numbers of summer and winter run) were placed into an insulated 100 L container filled with aerated river (fresh) water. The container was placed in a boat and slowly transported from the river mouth to Point Atkinson, 20 km west of the river mouth. Over the eight hour transport period, a pump was used to slowly increase the salinity of the water, such that the fish were in full strength seawater at the time of release and sampling (25‰). These fish were not released with any untagged fish. As in previous years, a single VR2 receiver located at the river mouth monitored movement out of the freshwater environment, and an additional VR2 was placed further out in the estuary to monitor estuarine movement of river released smolts.

Health assessments were performed on three groups of summer run steelhead; Group 1 – hatchery fish randomly sampled from their rearing channel; Group 2 - river (Swinburne) released fish (FW: River) sampled from the river immediately after passing through the hose; and Group 3 - marine released fish (SW: Marine) sampled from the transport container at the Centre for Aquaculture and Environmental Research (CAER) dock, located in West Vancouver approximately 200 m from Pt. Atkinson (ca.<5 min prior to their release at Pt. Atkinson).

At each sample time, thirty untagged fish from each group were euthanised and sampled using the methods described below. The same parameters examined in the 2007 study were also examined in 2008, with two exceptions; plasma protein and respiratory burst activity were not measured in 2008. Serum cortisol was included in the 2008 health assessment to examine transport stress in the fish at the time of release. Another difference between the sampling performed in 2007 and 2008 (and 2009), was that pathogen testing changed to focus on the detection of a single pathogen, *Renibacterium salmoninarum* (causative agent of bacterial kidney disease, BKD). *R. salmoninarum* was found to be much more prevalent in 2007 than the other pathogens tested for. Resources were therefore dedicated to examine future released fish for the presence of the BKD pathogen. Other reasons for focusing on *R. salmoninarum* were related to the high prevalence of the pathogen being detected in ocean caught salmonids [19] and research that suggests that even low levels of prevalence could negatively impact ocean survival by impairing predator avoidance response [20] and because of the chronic nature of the disease [21].

#### 2009 Study

In 2009, a total of 150 summer run steelhead were tagged and randomly released with one of eight groups of untagged fish (163.6±21.2 mm). The different release groups are described in Table 1, and were designed to test the effect of release site (FW or SW), release protocol (FW mouth or river, SW barged or marine) and vaccination, on migratory behaviour and survival. The first FW group (37 tagged fish) was released on May 14th into the river at a site just above the river mouth (FW: Mouth). The second FW group (37 tagged fish) was released on May 21st into the river at the conventional Swinburne location (FW: River). Both freshwater releases were performed using truck transport. As in previous years, a single VR2 located in the river mouth monitored the movement of fish out of the river, and an additional VR2 in the estuary located at the 2008 site monitored estuarine migration. In 2009, two additional VR2 receivers were placed 1 km west of the river at the same position used in the 2006 study.

Both seawater releases were performed on May 20^th^ and involved loading two trucks, each with 38 tagged fish. One truck was driven onto a barge at a site located approximately 5 km east of the Seymour River mouth and slowly transported by the barge to the previous years' release site at Pt. Atkinson (SW: Barged). During this transport the freshwater was slowly replaced with seawater, so that by the time the 8 h transport was complete, the fish were in full strength seawater (25‰) at the time of release. The second truck drove to CAER (∼60 min drive from hatchery), where the fish were released directly into seawater (SW: Marine) from the transport truck.

Two subgroups were created within each of the four release groups, by vaccinating half the tagged fish against four common water-borne pathogens (the remaining half of the tagged fish were considered unvaccinated controls useful for comparisons with results from previous years releases). The vaccinations were performed at the Seymour River hatchery February 25^th^, by intraperitoneal injection with 0.1 mL of Advantigen®4.1 (Microtek International Inc., Saanichton, Canada). This vaccine was chosen because it contained components that would provide protection against the most prevalent bacterial diseases causing disease and mortality in the BC salmon farming industry: furunculosis caused by *Aeromonas salmonicida*, vibriosis caused by *Listonella anguillarum* (serotypes 1 and 2), and cold-water vibriosis caused by *Vibrio salmonicida*. The vaccinated fish were reared separately from the unvaccinated fish, until they were tagged and released (approximately 450 degree days post-vaccination).

Health assessments were performed on fish sampled at the hatchery prior to release, and from two of the released groups; FW: River and SW: Marine. These groups were selected for analyses, because it would allow a more direct comparison with previous years' results, as well as for logistical reasons related to fish accessibility. A total of 8 different groups of fish (12 fish/group) were sampled for these health assessments. The first two groups were the control fish randomly sampled at the hatchery one day before the SW releases; Group 1- Hatchery unvaccinated and Group 2 – Hatchery vaccinated. Sampling the vaccinated and unvaccinated transported fish posed some logistical problems, because of the difficulties associated with keeping the vaccinated fish separate from the unvaccinated fish in the transport tank. Therefore, it was decided to sample just the SW: Marine and FW: River released fish. In each of these two transports, two mesh buckets containing either vaccinated or unvaccinated fish (12 fish/bucket) were placed inside the transport tank along with the other tagged and untagged fish, and transported to the release site. The fish were placed in these buckets and transported to provide comparative data that could be used to determine if there were any differences between any of the health parameters between vaccinated and unvaccinated fish at the time of release. The health results of the bucket transported fish were therefore not used to interpret the survival and behaviour of the released fish because of this difference in transport treatment. At both the FW: River and SW: Marine releases, the buckets were removed from the truck tank and those fish immediately euthanised and sampled. These four groups (Groups 3–6) were therefore designated as; (Group 3) Bucket Sampled: FW: River-unvacc, (Group 4) FW: River-vacc, (Group 5) SW: Marine-unvacc, and (Group 6) SW: Marine-vacc. To obtain results that could be more directly comparable to previous years' results, unvaccinated fish were sampled immediately after passing through the transport hose and designated; Group 7 - Hose Released FW: River-unvacc, and Group 8 – Hose Released SW: Marine-vacc.

The 2009 health assessments included general health observations and measurements, hematology, smoltification status, and pathogen (*R. salmoninarum*) prevalence. Unlike previous years, blood electrolytes were not measured. To determine the potential efficacy of the vaccine administered to the released fish, serum antibody titres for two vaccine components (*L. anguillarum* and *A. salmonicida*) were included in the health assessment. These two antigens were selected for testing vaccine efficacy because they both cause diseases (vibriosis and furunculosis, respectively) known to be prevalent in salmon farmed throughout BC.

### Health and physiology analyses

Fish were starved 24–48 h prior to sampling. At each sample time, the fish were randomly selected and euthanised in a bath containing a lethal concentration of buffered (400 ppm sodium bicarbonate) TMS (200 ppm). All fish were quickly weighed (g), measured (fork length; mm) and condition factors (CF) later calculated (CF  =  weight (g)/fork length (cm)^3^ ×100). A necropsy based health assessment was performed on all fish using a modification of the method described by [22] and previously utilized to document health of hatchery and wild coho salmon (*Oncorhynchus kisutch*) [23]. Briefly, this method (described in Table S1) involved a visual external and internal examination of all tissues and organs. The necropsy score was based on a total score of 23, which included ranking the appearance of external (skin, eyes, gills, fins, pseudobranch) and internal organs (amount of visceral fat, kidney, liver, spleen, gall bladder, posterior intestine). A score of 0 represented a normal healthy appearing fish with no visceral fat stores, lesions, swellings, hemorrhage, discolorations, or any other signs of abnormalities.

Following the weight and length measurements, the tails were immediately severed using sterile blades, and blood collected for the following hematological analyses: hematocrit, hemoglobin, erythrocyte counts, differential leucocyte numbers, mean erythrocyte volume, mean erythrocytic hemoglobin [24]. Following the hematocrit measurements, the capillary tubes were broken and the plasma plus leucocrit layer placed onto cleaned glass welled slides. The slides were incubated in a humid chamber for 30–60 mins, and the respiratory burst activity of the attached peripheral blood leucocytes were determined using nitroblue tetrazolium (NBT) assay [25]. This glass adherent NBT assay provided data on the activity of the non-specific immune system, and was performed only in 2007. The remaining fresh blood was placed into a sterile propylene tube, left overnight at 5°C, then centrifuged (2,000 x g at 5°C, 5 min) to collect serum which was stored at −80°C. Serum was later analyzed for levels of sodium, potassium, calcium, chloride, glucose and lactate using a Stat Profile Plus 9 blood gas instrument (Nova Biomedical Corporation, MA, USA). Serum cortisol levels were measured in fish sampled in 2008 and 2009, using a commercially available cortisol enzyme linked immunosorbent assay (ELISA; Neogen Corporation, Lexington, KY), that has been validated for use in salmonids [26]. In 2009, the serum was tested for the presence of antibodies against *L. anguillarum* and *A. salmonicida* (two vaccine components) using the agglutination titre method [27]. During sampling, the left gill arches from each fish were removed, placed in foil and frozen in liquid nitrogen. The gill samples were stored at −80°C and later analyzed to determine Na/K-ATPase enzyme activity [28], [29]. Livers were removed, weighed, and with whole body weights used to calculate the hepatosomatic index (HSI, liver weight/whole body weight ×100). The kidney was aseptically removed, placed in sterile bags, frozen in liquid nitrogen and stored at −80°C until analyzed for the presence of *R. salmoninarum* using the polymerase chain reaction (in 2007, [30]) and/or enzyme linked immunosorbent assay (in 2008 and 2009, [31]) detection methods.

In 2007, the presence of two bacterial pathogens commonly found in salmon hatcheries - *A. salmonicida* (causative agent of furunculosis) and *Flavobacterium psychrophilum* (causative agent of bacterial gill disease) were determined using culture methods. In this regard, the spleen and kidney were aseptically swabbed and cultured onto tryptic soy agar to determine the prevalence of *A. salmonicida,* and gill tissue was swabbed and cultured onto tryptone yeast extract salt agar to determine the prevalence of *F. psychrophilum* (methods outlined in [32]). Bacterial growth was presumed to be these pathogens based on the presence of pigments (*A. salmonicida*), morphological characteristics and Gram stain results of re-isolated bacterial colonies.

### Data Analyses

Apparent survival rates were determined by assuming that a fish detected on sub-array “a” but not detected on the next sub-array “b” (or later on sub-array “c”) had died. In some instances, where fish had passed undetected past a receiver sub-array (i.e., when detected on sub-array “a” and “c” but not “b”) it was included and counted as a survivor. Detection efficiencies for the tags used in this study were in the p = 80∼100% range, so correcting for imperfect detection would result in increasing apparent survivals by no more than *p* ^−1^≤1.2 (≤20%). Because the focus of this project was to compare the relative survival of experimental release groups implanted with the same type of acoustic tag (within each year), the degradation in detection efficiency normally accounted for by the use of maximum likelihood Cormack-Jolly-Seber mark-recapture models (which estimate absolute survival) is less relevant because the goal of this study was to test whether the proportions of two (or more) tagged groups of fish had equal survival across the array.

We calculated apparent percent survival for FW released fish in 4 segments: (1) river or mouth (FW) release site to estuary, (2) estuary to NSOG, (3) NSOG to QCS, and (4) FW release site (mouth or river) to QCS. Similarly, survival of the SW released fish was determined for 3 segments; (1) Pt. Atkinson or CAER (SW) release site to NSOG, (2) NSOG to QCS, and (3) SW release site (Pt. Atkinson or CAER) to QCS. A summary of the segment survival data is reported in Table S5. Overall apparent survival was calculated as percent of fish detected on the QCS in relation to the total number of fish released. Binomial proportion tests (Fishers exact test and Chi square tests) were used to compare the apparent survival rates between the experimental release groups within each year. Within year comparisons between overall apparent survival included examining statistical comparison between summer run and winter run steelhead, day and night release, staggered release dates, vaccinated and unvaccinated, FW and SW released fish. Significant differences were noted where p<0.05.

The physiological data was analyzed for differences between the various treatment groups using Student t-tests, and One Way ANOVAs. Proportion data (hematocrit, respiratory burst activity) were arcsin transformed prior to statistical analyses. Significant differences were noted where p<0.05.

## Results

An overview of the detection and apparent survival data for all four years is presented in Table 2 and 3, respectively. The detailed results on the survival of each group of experimental fish between the different migration segments from release up to the QCS array is provided separately in Table S5.

**Table 2:** Overview of the detections (number of detections at each location noted, along with the percent of total number tagged) of the various release groups of tagged Seymour steelhead smolts from 2006 to 2009, on the Northern Strait of Georgia (NSOG), Queen Charlotte Strait (QCS), and Juan de Fuca (JDF) POST arrays.

	Total Released	Estuary Detections	NSOG Detections	QCS Detections	JDF Detections
2006					
FW: River - Day	25	19 (76%)	2 (8%)	1 (4%)	0
FW: River - Night	25	17 (68%)	3 (12%)	0	0
2007					
FW: River - Early	20	18 (90%)	1 (5%)	0	0
FW: River - Middle	20	14 (70%)	2 (10%)	1 (5%)	0
FW: River - Late	20	14 (70%)	3 (15%)	0	0
2008					
FW: River	30	18 (60%)	3 (10%)	1 (3%)	0
SW: Barged	30	na	7 (23%)	3 (10%)	1 (3%)
2009 *					
FW: River - Unvaccinated	19	10	1 (5%)	1 (5%)	0
FW: River - Vaccinated	18	17	3(17%)	3 (17%)	0
FW: Mouth - Unvaccinated	19	17	0	0	0
FW: Mouth - Vaccinated	18	16	0	0	0
SW: Barged - Unvaccinated	19	n/a	10 (53%)	5 (26%)	0
SW: Barged - Vaccinated	19	n/a	15 (79%)	6 (32%)	0
SW: Marine - Unvaccinated	19	n/a	7 (37%)	4 (21%)	0
SW: Marine - Vaccinated	19	n/a	11 (58%)	6 (32%)	0

There were no significant differences (p>0.05) between the summer run and winter run steelhead from 2006–2008, therefore results from each strain were combined.

*In 2009, the number presented in the NSOG Detections column, is the corrected number (i.e., it includes those fish that were undetected on the NSOG, but were later detected on the QCS line).

### 2006 Study

Table 2 provides an overview of the combined number of detections following the release of summer run (13 tagged fish/group) and winter run (12 tagged fish/group) steelhead released during the day or night of May 10, 2006. There were no differences in survival at any time between the summer run and winter run steelhead, nor any difference between the day and night releases (p>0.05). The migration route of these fish was through the Johnstone and Queen Charlotte Straits, with poor survival throughout the migration: 10% survival to NSOG and only 2% of the total number of tagged and released fish reached the QCS line (1/50 fish surviving).

### 2007 Study

Results from the 2007 study indicate (as was found in 2006) no differences between the summer run and winter run steelhead. Combined results (summer run and winter run) presented in Table 2, also show similar patterns of migration (via Johnston and Queen Charlotte Strait) and overall survival (2% or 1/60 survival rates) to those observed in 2007. The comparison of the three different experimental groups reveal no statistical difference (p>0.05) between the different release dates. In both 2006 and 2007, the FW survival of the river-released fish was significantly higher than in any of the SW segments (refer to Table 3).

**Table 3:** Comparison of the apparent percent survival of unvaccinated Seymour steelhead smolts for the various experimental groups released from 2006–2009.

	FW survival		SW survival		Overall survival
Release Groups	FW release site to estuary	Estuary to NSOG	SW release site to NSOG	NSOG to QCS	Release site to QCS
2006					
FW: River-Night	68% (17/25)	18% (3/17)	na	0% (0/3)	0% (0/25)
FW: River-Day	76% (19/25)	11% (2/19)	na	50% (1/2)	4% (1/25)
All FW	72% (36/50)**^a^**	14% (5/36)**^b^**	na	20% (1/5)**^b^**	2% (1/50)
2007					
FW: River-Early	90% (18/20)	6% (1/18)	na	0% (0/1)	0% (0/20)
FW: River-Middle	70% (14/20)	14% (2/14)	na	50% (1/2)	5% (1/20)
FW: River-Late	70% (14/20)	21% (3/14)	na	0% (0/3)	0% (0/20)
All FW	77% (46/60)**^a^**	13% (6/46)**^b^**	na	17% (1/6)**^b^**	2% (1/60)
2008					
(All) FW: River	60% (18/30)	17% (3/18)	na	33% (1/3)	3% (1/30)
(All) SW: Barged	na	na	20% (6/30)	50% (3/6)	10% (3/30)
2009					
FW: River	53% (10/19)	10% (1/10)	na	100% (1/1)	3% (1/19)
FW: Mouth	90% (17/19)	0% (0/17)	na	0% (0/0)	0% (0/19)
SW: Barged	na	na	53% (10/19)	50% (5/10)	26% (5/19)
SW: Marine	na	na	37% (7/19)	57% (4/7)	21% (4/19)
All FW	71% (27/38)	4% (1/27)	na	100% (1/1)	3% (1/38)
All SW	na	na	45% (17/38)	53% (9/17)	24% (9/38)

Percent survival was based on the number of detections between each segment of the migration from point of release, up to and including the POST arrays at the Northern Strait of Georgia (NSOG) and Queen Charlotte Strait (QCS). There were no significant differences (p>0.05) between the summer run and winter run steelhead from 2006-2008, therefore results from each strain were combined. Different letters within a row, indicate significant differences in survival were detected between the release segments for the combined FW release groups in 2006 and 2007(p<0.05).

The additional receivers used in 2007 demonstrated that some fish did not immediately swim out of Burrard Inlet into Georgia Strait, but migrated in the opposite direction to Indian Arm. These two fish (and eight others) were however later detected on the English Bay line (and later on the NSOG line). The English Bay detection data has not been presented because the array was disrupted (receiver lines were cut and receivers lost), and therefore detection efficiencies were degraded. However, the data suggests that mortality was higher immediately after ocean entry inside Burrard Inlet rather than later in the migration.

### 2008 Study

After analyzing the survival data from 2006 and 2007, it was apparent that early marine survival was likely being compromised either by an unknown biotic (predators, disease) or abiotic (contaminants) factor(s) within the estuary and/or Burrard Inlet. The focus of the 2008 study was therefore directed towards examining the survival of a group of fish released at the conventional site in the river (Swinburne), with a second group released in the marine environment at a location on the outskirts of Burrard Inlet (Point Atkinson). The study was therefore designed to tag and release half the fish into the ocean as described in the methods section and the other half were released into the river using conventional procedures. An overview of the detections and survival of these fish is provided in Tables 2 and 3 respectively.

As previously found in 2006 and 2007, there was no significant difference in apparent survival between the summer run and winter run steelhead (p>0.05). There was however, a substantial increase in the overall apparent survival to QCS of the SW released marine-barged group (10%) when compared to the FW river released group (3%).

In 2008, there was a single fish (the only one in the four year study) that migrated south to cross the Juan de Fuca (JDF) array. This fish was later detected at the Northern end of Vancouver Island on the Lippy Point array.

### 2009 Study

The increased survival of the seawater released group of fish in 2008 suggested that the survival of hatchery steelhead could be significantly improved by releasing fish beyond Burrard Inlet. Therefore, in 2009 additional SW release groups were included in the study to further test the hypothesis that releasing hatchery fish into the marine environment at a distance from the river mouth (and in the direction of their natural seaward migration route), would improve survival. In the 2009 study we also vaccinated half the steelhead smolts to examine the effect of both factors (vaccination and release location - FW and SW) on early marine survival.

The detection results for the eight experimental groups compared in 2009 are presented in Table 2. A total of 12 fish passed the NSOG line undetected (a problem which was unique to the 2009 study only). The detection and survival data presented in Tables 2 and 3 respectively, are the corrected number of fish that were assumed to have passed the NSOG array, based on their subsequent detection on the QCS array. The lowest overall survival in 2009 was in the FW: Mouth group of released fish that appeared to have experienced 100% mortality following their release, because no fish were detected on the NSOG array. The FW: River group had higher survival to the QCS array than the FW: Mouth group (3% vs 0%, respectively). The survival of the two different SW release groups were not significantly different from each other, despite the SW: Barged group having experienced a gradual transition to SW from FW, while the SW: Marine group was released directly into SW following their FW transport.

The effect of vaccination on the early marine survival of the steelhead was not as clear as the effect of release location. Tables 4 and 5 present the apparent survival data for the vaccinated and unvaccinated fish within each of the four experimental release groups. There was no statistically significant difference between the vaccinated and unvaccinated fish, however there was a clear trend for vaccinated fish to experience improved survival. A significant difference in survival between vaccinated and unvaccinated fish (p<0.05) was detected in the first and very brief phase of early FW migration (FW release site to the estuary). In this particular phase of their migration, survival was significantly improved in the vaccinated group of fish. Also of interest, each of the replicate release groups within Table 5 experienced remarkably similar survival levels, suggesting that relatively small release groups could be used to measure survival because survival probability was stable over time.

**Table 4:** Comparison of the effect of vaccination on apparent survival of the 2009 experimental release groups of Seymour steelhead smolts.

	FW Survival		SW Survival	
Release Groups	FW release site to estuary	Estuary to NSOG	SW release site to NSOG	NSOG to QCS
FW: River-Unvac.	53% (10/19)	10% (1/10)	na	100% (1/1)
FW: River-Vaccin.	94% (17/18)	18% (3/17)	na	100% (3/3)
FW: Mouth-Unvac.	90% (17/19)	0% (0/17)	na	0%
FW: Mouth-Vaccin	89% (16/18)	0% (0/16)	na	0%
SW: Barged-Unvac.	na	na	53% (10/19)	50% (5/10)
SW: Barged-Vaccin.	na	na	79% (15/19)	40% (6/15)
SW: Marine-Unvac.	na	na	37% (7/19)	57% (4/7)
SW: Marine-Vaccin.	na	na	58% (11/19)	55% (6/11)
All FW: Unvac.	71% (27/38)*	4% (1/27)	na	100% (1/1)
All FW: Vaccin.	92% (33/36)*	9% (3/33)	na	100% (3/3)
All SW: Unvac.	na	na	45% (17/38)	53% (9/17)
All SW: Vaccin.	na	na	68% (26/38)	46% (12/26)

Percent survival was based on the number of surviving between each segment of the migration from point of release, up to and including the POST arrays at the Northern Strait of Georgia (NSOG) and Queen Charlotte Strait (QCS).

*refers to significant difference (p<0.05) was detected between vaccinated and unvaccinated fish for that particular group and segment of migration.

**Table 5:** Examination of the effect of release location (freshwater or seawater) and vaccination (vaccinated or unvaccinated) on the percent apparent survival of the 2009 experimental release groups of Seymour steelhead smolts.

	Freshwater Released	Seawater Released	Totals
Unvaccinated	3% (1/38) *[5% (1/19) FW: River][0% (0/19) FW: Mouth]	24% (9/38) *[21% (4/19) SW: Marine][26% (5/19) SW: Barged]	13% (10/76)
Vaccinated	8% (3/36) *[17% (3/18) FW: River][0% (0/18) FW: Mouth]	32% (12/38) *[32% (6/19) SW: Marine][32% (6/19) SW: Barged]	20% (15/74)
Totals	5% (4/74) *	28% (21/76) *	

*refers to statistically significant different detected between the survival of the combined FW release groups and the combined SW release groups within each row (Chi square, p<0.05). No significant differences were detected between the vaccinated and unvaccinated groups (i.e., between rows).

Table 5 provides a summary of the overall survival of the different experimental groups and clearly demonstrates a significant improvement in early marine survival for SW released fish, when compared to the FW released fish (p<0.001).

### Health/Physiology

The results from these analyses are presented in Table S2 (2007), Table S3 (2008) and Table S4 (2009). Overall, the various hematological, immunological and physiological results were within published normal ranges [33], and suggest that in general, the fish were in good health. The smoltification parameters that were measured (i.e., gill Na/K ATPase activity) indicate that the fish were physiologically prepared to adapt to a marine environment. There were no clinical signs of disease in any of the fish analyzed, as carefully conducted necropsies did not detect any lesions or abnormalities of any kind. Bacteriological tests performed in 2006 suggest a very low level of commonly occurring bacteria (*A. salmonicida, F. psychrophilum*) were present in tissues of <10% of the fish. In 2007, *R. salmoninarum* was detected in low levels in all of the fish (100%) using PCR. In 2008 and 2009, ELISA testing of kidney tissues failed to detect the pathogen in any fish.

## Discussion

In an effort to understand and mitigate increasingly poor adult returns of steelhead, we conducted a four year study using the Seymour River stock to compare the early marine survival and migration of hatchery-reared fish released using various experimental strategies. The pilot-scale POST array and temporary acoustic receivers were used to monitor and track tagged fish that were released at different times and locations. Results indicate that release timing (varying time of day or dates by several days) did not have a significant impact on early marine survival.

In contrast, marine survival to the QCS telemetry sub-array increased from just 2% in 2006 to 32% in 2009 when vaccinated fish were transported for release into the ocean (compared with conventional releases of unvaccinated fish direct into the river; which remained consistently low across all years of the study). This increase in survival was repeated in both 2008 and 2009, and occurred regardless of whether the fish were gradually acclimated to seawater (2009 SW: Barged and 2008 SW: Barged) or directly placed into seawater from the freshwater transport tank (2009 SW: Marine). It is unlikely that, given the long intervening period of ocean life (and the variable number of years juveniles spend at sea) that such a clear result would be obtained if all of the subsequent ocean variation affecting adult returns was also included in the analysis. As a practical point, it would also require an additional 4 years after the end of the 2009 smolt migration before an analysis could be completed. The use of the POST telemetry array thus both shortened the research cycle and allowed sharper focus on the early marine phase.

In 2009, two different methods of releasing fish into the SW environment were compared (SW: Marine and SW: Barged). There were no significant differences in overall survival between these two groups (p = 0.944), indicating that the stress involved in releasing fish directly from freshwater to seawater did not compromise early marine survival. Increased cortisol levels were found in the transported fish however this was likely related to the increased interrenal responsiveness of the smoltification process [34]. Indeed, all the fish sampled prior to being released appeared to be physiologically prepared for the osmotic challenges associated with the transfer to seawater (as indicated by the high gill Na^+^K^+^-ATPase activities).

Releasing fish directly into the river (conventional practice) resulted in QCS survival rates that were consistently very low (approximately 3%) in each of the four years of this study. In an attempt to improve the survival of FW released fish, a second FW release group was included in the 2009 comparison study (FW: Mouth). The FW: Mouth group of fish were released at the river mouth (approximately 100 m from the estuary receivers) in the afternoon just above tidewater, on a date with a large high tide expected around midnight. This release date and location was chosen in an attempt to increase survival of these fish by facilitating their entrance to seawater in the dark at a high tide (conditions thought to encourage the migration of the fish out of the river and estuary, while reducing the risk of predation). However, the survival of this group was the lowest of any group in the study, with no fish detected on the NSOG or QCS line. It is unknown why the FW: Mouth released fish experienced such high mortality, but they were released one week earlier than the FW: River fish, which may have affected by factors such as food availability. Shifting climate regimes and ocean conditions may be altering the timing of plankton blooms, such that released smolts do not have sufficient prey items to support the nutritional and energetic needs required to support early marine survival [21]. Another possible cause of early marine mortality might be related to the presence of contaminants in the Seymour River estuary and Burrard Inlet. Smolts released into the river or river mouth area must pass through an industrialized urban area that likely contains a mixture of toxicants, which might be compromising fish health and increasing their susceptibility to disease [35], [36]. It is also possible that the river estuary contained an unusually high number of predators (birds, fish, seals, etc). Predation risk is considered to be extremely high the first few days of seawater exposure, as the fish tend to swim near the surface where salinities are often lower [37]. Subclinical BKD infections may also increase predation risk [20]. BKD was detected by PCR in 2007, but not by ELISA in 2008 and 2009. It is possible that low levels of *R.salmoninarum* were present in the fish in 2008 and 2009, which were not detected due to the relative insensitivity of the ELISA relative to the PCR test.

The low apparent survival of FW released fish may in part be attributed to residualisation of the fish in the river after release. The issue of steelhead residualisation has been studied in both the Englishman and Cheakamus rivers [38], and results suggest that residualisation is minimal in these rivers. In the Seymour River, re-capture of adipose clipped hatchery steelhead smolts found above the Seymour Canyon (2 km upstream from the release site) in a rotary screw trap suggest that in 2009, perhaps 8∼9% of the released smolts may have residualised (Don McCubbing, InStream Fisheries Research Inc ., 1698 Platt Crescent, North Vancouver BC V7J 1Y1; pers. comm.). Although not tagged with acoustic tags, these fish could have only come from one of the two FW release groups.

Our study provides not only data on marine survival of released steelhead, but also new data on marine migratory behavior. For example, in 2006 the additional temporary receivers placed in Indian Arm detected four fish after release, with two of these fish later detected on the English Bay line. Our finding a small proportion of fish navigated the “wrong” way in the sense that they went up a blind channel and then in some cases reversed course, is consistent with the pattern seen in steelhead from the geographically similar Waukwass River [39]. In 2008 a single fish from the SW: Barged group was detected on the JDF line (the only detection of a tagged Seymour smolt taking the southern exit from the Strait of Georgia in the entire four years of this project). This fish was subsequently detected on the Lippy Point Line off the Northwest tip of Vancouver Island, so northward migration over the continental shelf continued after exiting the Strait of Juan de Fuca. In addition, eight fish from this group were detected on a temporary sub-array in nearby Howe Sound. These results suggest that there may be some initial straying associated with marine releases, as the normal pattern of marine migration (rapid movement out of the river, through Burrard Inlet and north up to QCS and the open Pacific) was not observed for all fish.

In all four years of the study, the migration route followed by the Seymour steelhead (via Johnstone and Queen Charlotte Straits rather than Juan de Fuca Strait) is identical to that followed by steelhead from the Cheakamus and Englishman systems [9]. In contrast, steelhead from the Cowichan River in Southeast Vancouver Island and from streams that flow into Puget Sound migrate consistently through Juan de Fuca Strait to the Pacific. It is possible that steelhead from specific populations in the southern Strait of Georgia and Puget Sound have distinct migration pathways or that the Fraser River plume plays an important factor in determining the migration pattern of the more southern populations. Whatever the specific cause, the consistency of the choice of either the northern or southern exit route by specific steelhead populations suggests that this behaviour is likely genetically determined and may be strongly shaped by evolutionary selection.

Hatchery-borne subclinical infections due to bacteria such as *A. salmonicida*, may have contributed to the poor marine survival of the released fish. Likewise, exposure to pathogens in the marine environment (e.g., *A. salmonicida, L. anguillarum*) could have also contributed to marine mortality. The vaccination results, which show enhanced survival when vaccinated fish were released, indicate that exposure to pathogens whether in the hatchery or marine environment may play a role in the poor marine survival, because there was a significant improvement in survival (p<0.001) in the freshwater environment in vaccinated fish (Table S5) and an increase (statistically insignificant) in early marine survival to QCS in vaccinated fish from all the release groups in 2009. Future research is needed to further investigate the effect of vaccination on the survival of wild salmon and validate these pilot-scale results.

Details of all the health results obtained from 2007–2009 are provided in Table S2 (2007), Table S3 (2008) and Table S4 (2009). In all years, fish appeared to be in good health with blood chemistry values and hematological values within the normal range [24], [33]. All fish showed high levels of Na^+^K^+^ - ATPase activity in gill tissue which indicated that the smolts were physiologically prepared to adapt to a marine environment [40], [41]. There were few differences between groups within each year, and few differences between years. The primary difference between groups was related to body size. In 2007, the summer run steelhead were significantly larger than the winter run steelhead. Another size difference was noted between vaccinated and unvaccinated fish sampled in the hatchery in 2009. The vaccinated fish were smaller which may be the result of reduced appetite following vaccination. This temporary reaction has been reported previously in Atlantic salmon (*Salmo salar*) [42].

In general, the size of the fish that were tagged (Table 1), untagged (refer to Methods section) and sampled for physiological parameters (Tables S2, S3, S4) were approximately equal. The use of the larger high acoustic power tags (V9-1H) in 2006, made it necessary to use slightly larger fish. However, in 2006 and each subsequent year, the maximum tag burden was estimated to be less than 5.3%. The absence of any post-surgical mortality indicates that using acoustic tags did not compromise the survival of the released fish, which is consistent with studies on chinook, coho, and steelhead that when properly done, surgical implantation can result in very low rates of mortality, tag shedding, and yield survival rates similar to those of smolts tagged with much smaller PIT tags [12], [43], [44]. The travel times for each group (not presented) were also similar with FW ranges in the order of 0.67–1.0 body lengths/second (12.9–16.5 km/d). Marine migration speeds were slightly faster at 1.0–1.3 body lengths/second (15.8–19.4 km/d).

The approximate detection efficiency of the tags used in this study was 100% in 2006 and approximately 90% in 2007–2009, with the variation between years primarily attributable to the use of high acoustic power tags in 2006. The differences in detection efficiency and/or tag type used between years does not however, have any effect on the conclusions from this study because the goal of this study was to compare different release strategies within a single year using a common tag type.

In summary, our results do not yet explain why marine survival is so low for southern British Columbia steelhead populations, but does however provide some new insight into possible locations associated with high mortality. Although some of the possible reasons for mortality may be local to the Seymour River (i.e., industrial pollution and/or predators at the river mouth) the broad geographic expanse of salmon populations experiencing low marine survival, indicate a more general problem that also affects many other steelhead populations. The results of this study suggest that early marine survival can be significantly improved by releasing fish directly into the marine environment rather than the river (i.e., avoiding the estuary and nearshore coastal environment). Time of day (day or night), release date (plus or minus 10 days), and release method (direct release or gradual acclimation) did not have a significant effect on early marine survival. Vaccination however, does appear to contribute to improved survival raising the question of whether or not a disease agent has spread over time and become more prevalent. Finally, our multi-year study demonstrated that acoustic telemetry can provide timely and revealing results that can be used by salmon enhancement hatcheries to rapidly improve our understanding of the factors influencing the marine survival of smolts in the earliest phase of their ocean life. It is hoped that this knowledge can be used to improve the cost efficiency and husbandry practices of hatcheries, increase adult return rates and recreational angling opportunities. This is important because some steelhead stocks are now considered to be no longer self-sustaining due to profound declines in marine survival that have occurred in the last two decades [1].

## Supporting Information

Table S1 Description of scoring system for the necropsy based health assessments performed on the Seymour steelhead smolts sampled in 2007–2009. The higher score indicates a greater disparity from the appearance of those tissues from normal tissues (i.e., a score of 0 indicates a completely normal healthy appearing fish, while a score of 23 indicates a fish that appears abnormal and unhealthy in every respect).
(0.05 MB DOC)

Table S2 Summary of results following the 2007 health assessment sampling of Seymour River steelhead. Fish were sampled at the Seymour Hatchery prior to release. Results are expressed as mean standard error (n = 30 per group). * indicate significant differences between the groups for a particular parameter.
(0.06 MB DOC)

Table S3 Summary of results following the 2008 health assessment of Seymour River summer steelhead. River release (FW) fish were sampled at the point of release into the Seymour River; Hatchery fish were sampled under resting conditions at the Seymour Hatchery; Marine Release (SW) fish were sampled following a 6 hour transport, at the point of release (13°C, 25‰ salinity). Results are expressed as mean standard error (n = 30 per group). Different letters indicate significant differences between the groups for a particular parameter.
(0.07 MB DOC)

Table S4 Summary of results following the 2009 health assessment of summer steelhead. Eight groups of fish were sampled (n = 12 per group, except pathogen prevalence testing which was performed on 30 per group). Vaccinated and unvaccinated fish were sampled at the hatchery, and from buckets placed into the transport tank. Unvaccinated fish were also sampled from the river and ocean immediately following passage through the transport pipe at the time of release. Results are expressed as mean standard error. Different letters indicate significant differences between the groups for a particular parameter.
(0.07 MB DOC)

Table S5 Summary of survival data of the various treatment groups of Seymour steelhead released from 2006–2009. Percent survival was based on the ratio of survivors to total number per designated segment of the migration up to and including the Northern Strait of Georgia (NSOG) and Queen Charlotte Strait (QCS). Overall survival from the river release site to the estuary was higher than other segments of the migration in 2006 and 2007.
(0.08 MB DOC)
