# Supplementary material for: The Effect of Hatchery Release Strategy on Marine Migratory Behaviour and Apparent Survival of Seymour River Steelhead Smolts (Oncorhynchus mykiss)
Source: PLoS One. 2011 Mar 29;6(3):e14779. doi: 10.1371/journal.pone.0014779 (PMC3066170; doi:10.1371/journal.pone.0014779)
Supplement: Table S1 — Description of scoring system for the necropsy based health assessments performed on the Seymour steelhead smolts sampled in 2007–2009. The higher score indicates a greater disparity from the appearance of those tissues from normal tissues (i.e., a score of 0 indicates a completely normal healthy appearing fish, while a score of 23 indicates a fish that appears abnormal and unhealthy in every respect). (0.05 MB DOC) [file pone.0014779.s001.doc]

| Tissue / Organ | Observation details | Score |
| --- | --- | --- |
| Extremities | Fins/skin -parr marks, silvering, lesions, hemorrhage | 0-3 |
| Gills | Colour, parasites, fraying | 0-2 |
| Pseudobranch | Colour, swelling | 0-2 |
| Eyes | Swelling, cataracts, hemorrhage | 0-2 |
| Liver | Size, colour, lesions | 0-2 |
| Kidney | Size, colour, lesions | 0-2 |
| Spleen | Size, colour, lesions | 0-2 |
| Visceral Fat | Extent of coverage from intestines to pyloric caeca | 0-4 |
| GI Tract hemorrhage | Hemorrhagic areas surrounding posterior intestine | 0-2 |
| Gall Bladder | Size, bile amount and colour | 0-2 |
