# Supplementary material for: The Effect of Hatchery Release Strategy on Marine Migratory Behaviour and Apparent Survival of Seymour River Steelhead Smolts (Oncorhynchus mykiss)
Source: PLoS One. 2011 Mar 29;6(3):e14779. doi: 10.1371/journal.pone.0014779 (PMC3066170; doi:10.1371/journal.pone.0014779)
Supplement: Table S2 — Summary of results following the 2007 health assessment sampling of Seymour River steelhead. Fish were sampled at the Seymour Hatchery prior to release. Results are expressed as mean standard error (n = 30 per group). * indicate significant differences between the groups for a particular parameter. (0.06 MB DOC) [file pone.0014779.s002.doc]

|  | Summer steelhead | Winter steelhead |
| --- | --- | --- |
| *General Health Observations:* |  |  |
| Weight (g) | 66.52 ± 2.92 * | 57.67 ± 2.72 |
| Fork Length (mm) | 188 ± 2.4 | 181 ± 2.6 |
| Condition Factor (K) | 0.99 ± 0.01 * | 0.95 ± 0.01 |
| Hepatosomatic Index (%) | 0.65 ± 0.020 | 0.60± 0.017 |
| Necropsy Based Health Score | 6.23 ± 0.11 | 6.10 ± 0.14 |
| *Hematology:* |  |  |
| Respiratory Burst Activity (% NBT pos.) | 0.25 ± .089 | 0.21 ± 0.073 |
| Leucocyte Nos. (x 10^4^/mL) | 2.06 ± 0.16 | 2.26 ± 0.26 |
| Erythrocyte Nos. (x 10^6^/mL) | 1.63 ± 0.025 | 1.61 ± 0.030 |
| Hematocrit (%) | 50.2 ± 0.81 * | 47.2 ± 0.72 |
| Hemoglobin (g/dL) | 6.49 ± 0.089 | 6.58 ± 0.093 |
| Mean Erythrocyte Volume (x 10^-15^ L) | 309 ± 5.33 | 296 ± 6.33 |
| Mean Erythrocytic Hemoglobin (x 10^-15^g) | 40.0 ± 0.58 | 41.3 ± 0.92 |
| Mean Erythrocytic Hemoglobin Content (g/dL) | 13.0 ± 0.19 * | 14.0 ± 0.24 |
| Plasma Protein (g/dL) | 3.61 ± 0.055 * | 3.35 ± 0.051 |
| Plasma Sodium (mEq/L) | 127.1 ± 5.28 * | 107.0 ± 6.54 |
| Plasma Potassium (mEq/L) | 0.524 ± 0.015 | 0.549 ± 0.016 |
| Plasma Chloride (mEq/L) | 106.7 ± 4.05 * | 93.44 ± 4.15 |
| Plasma Calcium (mEq/L) | 1.34 ± 0.061 * | 1.17 ± 0.054 |
| Plasma Lactate (mg/dL) | 8.11 ± 0.909 | 5.86 ± 0.268 |
| *Smoltification Indicators:* |  |  |
| Plasma Glucose (mg/dL) | 9.66 ± 0.499 | 8.54 ± 0.472 |
| Gill Na/K ATPase Activity (μM ADP/mg protein/h) | 8.47 ± 0.72 | 8.02 ± 0.56 |
| *Pathogen Prevalence:* |  |  |
| *Renibacterium salmoninarum* (PCR) | 100 % | 100 % |
| *Aeromonas Salmonicida* (culture) | 3 % | 0 % |
| *Flavobacterium psychrophilum* (culture) | 10 % | 10 % |
