# Supplementary material for: The Effect of Hatchery Release Strategy on Marine Migratory Behaviour and Apparent Survival of Seymour River Steelhead Smolts (Oncorhynchus mykiss)
Source: PLoS One. 2011 Mar 29;6(3):e14779. doi: 10.1371/journal.pone.0014779 (PMC3066170; doi:10.1371/journal.pone.0014779)
Supplement: Table S3 — Summary of results following the 2008 health assessment of Seymour River summer steelhead. River release (FW) fish were sampled at the point of release into the Seymour River; Hatchery fish were sampled under resting conditions at the Seymour Hatchery; Marine Release (SW) fish were sampled following a 6 hour transport, at the point of release (13°C, 25‰ salinity). Results are expressed as mean standard error (n = 30 per group). Different letters indicate significant differences between the groups for a particular parameter. (0.07 MB DOC) [file pone.0014779.s003.doc]

|  | FW Release | Hatchery Control | SW Release |
| --- | --- | --- | --- |
| *General Health Observations:* |  |  |  |
| Weight (g) | 55.8 ± 2.1 ^ab^ | 49.7 ± 1.9 ^a^ | 67.3 ± 4.2 ^b^ |
| Fork Length (mm) | 176 ± 2.0 ^a^ | 170 ± 13 ^a^ | 190 ± 4.5 ^b^ |
| Condition Factor (K) | 1.01 ± 0.067 ^a^ | 1.00 ± 0.054 ^a^ | 0.93 ± 0.0068 ^b^ |
| Standard Length (cm) | 16.0 ± 0.19 ^a^ | 15.5 ± 0.21 ^a^ | 17.2 ± 0.41 ^b^ |
| Peduncle Depth (cm) | 1.53 ± 0.032 ^a^ | 1.45 ± 0.023 ^a^ | 1.61 ± 0.046 ^b^ |
| Hepatosomatic Index (%) | 1.01 ± 0.025 ^a^ | 0.85 ± 0.13 ^b^ | 0.82 ± 0.021 ^b^ |
| Splenosomatic Index (%) | 0.058 ± 0.0047 ^a^ | 0.043 ± 0.0056 ^a^ | 0.056 ± 0.0034 ^a^ |
| Necropsy Based Health Score | 4.43 ± 0.164 ^a^ | 5.40 ± 0.113 ^b^ | 5.15 ± 0.129 ^b^ |
| *Hematology:* |  |  |  |
| Leucocyte Nos. (x 10^4^/mL) | 6.75 ± 0.48 ^a^ | 7.05 ± 0.24 ^a^ | 4.81 ± 0.31 ^b^ |
| Erythrocyte Nos. (x 10^6^/mL) | 1.19 ± 0.035 ^a^ | 1.40 ± 0.025 ^b^ | 1.20 ± 0.023 ^a^ |
| Hematocrit (%) | 38.3 ± 1.18 ^a^ | 39.5 ± 0.86 ^a^ | 39.6 ± 0.92 ^a^ |
| Hemoglobin (g/dL) | 9.74 ± 0.22 ^ab^ | 10.4 ± 0.27 ^a^ | 9.33 ± 0.22 ^b^ |
| Mean Erythrocyte Volume (x 10^-15^ L) | 332 ± 15.6 ^a^ | 285 ± 7.11 ^b^ | 334 ± 9.95 ^a^ |
| Mean Erythrocytic Hemoglobin (x 10^-15^g) | 82.7 ± 1.70 ^a^ | 74.6 ± 1.65 ^b^ | 78.3 ± 1.71 ^ab^ |
| Mean Erythrocytic Hemoglobin Content (g/dL) | 26.5 ± 1.47 ^a^ | 26.7 ± 0.99 ^a^ | 23.9 ± 0.72 ^a^ |
| White Blood Cell Nos. (x 10^4^/mL) | 6.75 ± 0.48 ^a^ | 7.05 ± 0.24 ^a^ | 4.81 ± 0.31 ^b^ |
| Plasma Sodium (mEq/L) | 165 ± 2.4 ^a^ | 153 ± 0.84 ^b^ | 170 ± 3.81 ^a^ |
| Plasma Potassium (mEq/L) | 0.90 ± 0.075 ^a^ | 0.95 ± 0.044 ^a^ | 1.07 ± 0.19 ^a^ |
| Plasma Chloride (mEq/L) | 139 ± 2.96 ^a^ | 127 ± 1.58 ^a^ | 139 ± 5.66 ^a^ |
| Plasma Calcium (mEq/L) | 1.14 ± 0.049 ^a^ | 1.26 ± 0.025 ^a^ | 0.93 ± 0.071 ^b^ |
| Plasma Lactate (mg/dL) | 8.80 ± 0.58 ^a^ | 8.66 ± 0.57 ^a^ | 10.1 ± 0.90 ^a^ |
| *Smoltification Indicators:* |  |  |  |
| Gill Na/K ATPase (μM ADP/mg protein/h) | 15.9 ± 1.5 ^a^ | 16.8 ± 1.9 ^a^ | 22.7 ± 2.2 ^b^ |
| Plasma Glucose (mg/dL) | 107.5 ± 11.0 ^a^ | 118.8 ± 15.8 ^a^ | 106.7 ± 10.3 ^a^ |
| Plasma Cortisol (ng/mL) | 92.9 ± 12.8 ^a^ | 44.7 ± 7.57 ^b^ | 45.6 ± 6.00 ^b^ |
| *Pathogen Prevalence:* |  |  |  |
| *Renibacterium salmoninarum* (ELISA) | nm | 0 % | nm |
