# Supplementary material for: The Effect of Hatchery Release Strategy on Marine Migratory Behaviour and Apparent Survival of Seymour River Steelhead Smolts (Oncorhynchus mykiss)
Source: PLoS One. 2011 Mar 29;6(3):e14779. doi: 10.1371/journal.pone.0014779 (PMC3066170; doi:10.1371/journal.pone.0014779)
Supplement: Table S4 — Summary of results following the 2009 health assessment of summer steelhead. Eight groups of fish were sampled (n = 12 per group, except pathogen prevalence testing which was performed on 30 per group). Vaccinated and unvaccinated fish were sampled at the hatchery, and from buckets placed into the transport tank. Unvaccinated fish were also sampled from the river and ocean immediately following passage through the transport pipe at the time of release. Results are expressed as mean standard error. Different letters indicate significant differences between the groups for a particular parameter. (0.07 MB DOC) [file pone.0014779.s004.doc]

|  |  | | | | SW: Marine Sample | | | | | | | FW: River Sample | | | | |
| --- | --- | --- | --- | --- | --- | --- | --- | --- | --- | --- | --- | --- | --- | --- | --- | --- |
|  | Hatchery Sample | | | | Pipe Released | | | Bucket Sampled | | | | Pipe Released | | Bucket Sampled | | |
|  | Vaccinated | | Unvaccin. | | Unvaccin. | | | Unvaccin. | | Vaccinated | | Unvaccin. | | Unvaccin. | | Vaccinated |
| *General Health Observations:* | | | | | | | | | | | | | | | | |
| Weight  (g) | 40.0 ± 2.52^ab^ | | | 50.8 ±  2.67^a^ | | 47.8 ± 4.17^ab^ | 47.0 ± 2.51^ab^ | | 49.5 ± 2.86^ab^ | | 52.6 ±  5.06^a^ | | 34.2 ±  3.78^b^ | | 49.8 ±  3.13^a^ | |
| Fork Length  (mm) | 170 ± 3.5^ab^ | | | 178 ±  3.0^a^ | | 166 ± 6.3^ab^ | 173 ± 3.3^ab^ | | 176 ±  3.3^a^ | | 174 ±  4.6^a^ | | 154 ±  6.3^b^ | | 176 ±  3.7^a^ | |
| Condition Factor  (g/(cm)^3^ x 100) | 0.81 ±  0.04^a^ | | | 0.90 ± 0.01^ab^ | | 1.01 ± 0.016^c^ | 0.91 ± 0.02^ab^ | | 0.89 ± 0.01^ab^ | | 0.98 ± 0.03^bc^ | | 0.91 ±\0.01^ab^ | | 0.90 ± 0.01^ab^ | |
| Hepatosomatic Index (%) | 1.00 ±  0.06^a^ | | | 0.78 ± 0.03^ab^ | | 0.96 ± 0.08^ab^ | 0.68 ±  0.07^b^ | | 0.77 ± 0.05^ab^ | | 0.73 ±  0.05^b^ | | 0.75 ±  0.03^b^ | | 0.74 ±  0.02^b^ | |
| Necropsy Based Health Score | 4.54 ± 0.31^ab^ | | | 3.71 ± 0.35^ab^ | | 3.46 ± 0.36^ab^ | 3.75 ± 0.28^ab^ | | 4.92 ±  0.34^a^ | | 3.08 ±  0.43^b^ | | 3.50 ± 0.31^ab^ | | 4.42 ± 0.34^ab^ | |
| *Hematology:* | | | | | | | | | | | | | | | | |
| Leucocyte Nos.  (x 10^4^/mL) | 2.65 ±  0.31 ^a^ | | | 2.97 ±  0.55 ^a^ | | 3.86 ±  0.82 ^a^ | 3.56 ±  0.52 ^a^ | | 3.94 ±  0.40 ^a^ | | 5.45 ±  1.12 ^a^ | | not measured | | not measured | |
| Erythrocyte Nos.  (x 10^6^/mL) | 1.37 ± 0.07^ab^ | | | 1.43 ± 0.07^ab^ | | 1.19 ±  0.07^a^ | 1.54 ±  0.04^b^ | | 1.44 ±  0.07 ^ab^ | | 1.48 ± 0.07^ab^ | | 1.37 ± 0.05^ab^ | | 1.44 ± 0.06^ab^ | |
| Hematocrit  (%) | 43.5 ±  1.73^a^ | | | 45.8 ±  0.56^a^ | | 50.3 ± 2.19^ab^ | 50.5 ±  0.67^b^ | | 46.0 ± 0.83^ab^ | | 51.1 ±  1.28^b^ | | 50.8 ±  1.05^b^ | | 48.0 ± 1.19^ab^ | |
| Hemoglobin  (g/dL) | 11.7 ±  0.37^a^ | | | 13.8 ± 0.28^bc^ | | 12.0 ± .057^ab^ | 14.1 ±  0.28^c^ | | 12.0 ± 0.53^ab^ | | 12.6 ±0.31^abc^ | | 13.5 ± 0.48^bc^ | | 12.9 ± 0.36^abc^ | |
| MEV *  (x 10^-15^ L) | 322 ±  11.7 ^a^ | | | 333 ±  21.9 ^a^ | | 438 ±  33.7 ^a^ | 331 ±  7.98 ^a^ | | 328 ±  17.5 ^a^ | | 349 ±  10.5 ^a^ | | 376 ±  16.1 ^a^ | | 339 ±  13.1 ^a^ | |
| MEH *  (x 10^-15^ g) | 86.9 ±  3.76 ^a^ | | | 99.2 ±  5.12 ^a^ | | 106.8 ±  6.03 ^a^ | 92.6 ±  2.19 ^a^ | | 85.9 ±  6.23 ^a^ | | 86.3 ±  2.60 ^a^ | | 101.0 ±  3.21 ^a^ | | 91.1 ±  3.95 ^a^ | |
| MEHC *  (g/dL) | 27.0 ± 0.74^ab^ | | | 30.0 ±  0.57^a^ | | 25.1 ±  1.38^b^ | 28.0 ±  0.44^a^ | | 26.1 ± 1.10^ab^ | | 24.8 ±  0.32^b^ | | 26.9 ± 1.02^ab^ | | 26.9 ± 0.54^ab^ | |
| *Smoltification Indicators:* | | | | | | | | | | | | | | | | |
| Na/K-ATPase (μM ADP/mg protein/h) | | 6.70 ±  1.08 ^a^ | | 9.31 ±  0.40 ^a^ | | 8.67 ±  1.19 ^a^ | 9.58 ±  0.69 ^a^ | | 8.05 ±  0.71 ^a^ | | 8.37 ±  0.64 ^a^ | | 9.22 ±  1.33 ^a^ | | 8.28 ±  1.11 ^a^ | |
| Plasma cortisol  (ng/mL) | | 13.2 ±  4.46^a^ | | 5.15 ±  2.28^a^ | | 50.3 ± 12.0^ab^ | 117.2 ± 12.9^b^ | | 108.8 ± 15.9^b^ | | 52.5 ± 9.50^ab^ | | 77.7 ± 15.0^ab^ | | 111.6 ± 10.9^b^ | |
| *Pathogen Prevalence:* | | | |  | |  |  | |  | |  | |  | |  | |
| *R.salmoninarum* | 0 % ^a^ | | | 0 % ^a^ | | not measured | not measured | | not measured | | not measured | | not measured | | not measured | |

* MEV (Mean Erythrocyte Volume); MEH (Mean Erythrocytic Hemoglobin); MEHC (Mean Erythrocytic Hemoglobin Content) cytic Hemoglobin Content)
