# Supplementary material for: The Effect of Hatchery Release Strategy on Marine Migratory Behaviour and Apparent Survival of Seymour River Steelhead Smolts (Oncorhynchus mykiss)
Source: PLoS One. 2011 Mar 29;6(3):e14779. doi: 10.1371/journal.pone.0014779 (PMC3066170; doi:10.1371/journal.pone.0014779)
Supplement: Table S5 — Summary of survival data of the various treatment groups of Seymour steelhead released from 2006–2009. Percent survival was based on the ratio of survivors to total number per designated segment of the migration up to and including the Northern Strait of Georgia (NSOG) and Queen Charlotte Strait (QCS). Overall survival from the river release site to the estuary was higher than other segments of the migration in 2006 and 2007. (0.08 MB DOC) [file pone.0014779.s005.doc]

|  |  |  | FW SURVIVAL | | SW SURVIVAL | | | OVERALL: Release site to QCS | | | |
| --- | --- | --- | --- | --- | --- | --- | --- | --- | --- | --- | --- |
| Year | Release Group | Strain/Treatment | River to Estuary | Mouth to Estuary | Estuary to NSOG | Pt Atkinson to NSOG | NSOG to QCS | River to QCS | Mouth to  QCS | FW: (River+Mouth) to QCS | SW: Pt Atkinson to QCS |
| 2006 | Night | Summer | 76.9% (10/13) | na | 20.0% (2/10) | na | 0% (0/2) | 0% | na | na | na |
|  | Night | Winter | 58.3% (7/12) | na | 14.3% (1/7) | na | 0% (0/1) | 0% | na | na | na |
|  | Day | Summer | 84.6% (11/13) | na | 18.2% (2/11) | na | 50.0% (1/2) | 7.69% (1/13) | na | na | na |
|  | Day | Winter | 66.7% (8/12) | na | 0% (0/8) | na | 0% | 0% | na | na | na |
| 2007 | Early | Summer | 90% (9/10) | na | 0% (0/9) | na | 0% (0/0) | 0% | na | na | na |
|  | Early | Winter | 90% (9/10) | na | 11.1% (1/9) | na | 0% (0/1) | 0% | na | na | na |
|  | Normal | Summer | 60% (6/10) | na | 16.7% (1/6) | na | 100% (1/1) | 10.0% (1/10) | na | na | na |
|  | Normal | Winter | 80% (8/10) | na | 12.5 % (1/8) | na | 0% (0/1) | 0% | na | na | na |
|  | Late | Summer | 80% (8/10) | na | 25.0% (2/8) | na | 0% (0/2) | 0% | na | na | na |
|  | Late | Winter | 60% (6/10) | na | 16.7% (1/6) | na | 0% (0/1) | 0% | na | na | na |
| 2008 | River | Summer | 60% (9/15) | na | 11.1% (1/9) | na | 100% (1/1) | 6.7% (1/15) | na | na | na |
|  | River | Winter | 60% (9/15) | na | 22.2% (2/9) | na | 0% (0/2) | 0% (0/15) | na | na | na |
|  | Marine Barged | Summer | na | na | na | 26.7% (4/15) | 75% (3/4) | na | na | na | 20% (3/15) |
|  | Marine Barged | Winter | na | na | na | 13.3% (2/15) | 0% (0/2) | na | na | na | 0% (0/15) |
| 2009 | River -Vacc. | Summer | 94.4% (17/18) | na | 17.6% (3/17) | na | 100% (3/3) | 16.7% (3/18) | na | na | na |
|  | River –Unvac. | Summer | 52.6% (10/19) | na | 10.0% (1/10) | na | 100% (1/1) | 5.26% (1/19) | na | na | na |
|  | Mouth – Vacc. | Summer | na | 88.9% (16/18) | 0% (0/16) | na | 0% |  | 0% (0/18) | na | na |
|  | Mouth – Unvac. | Summer | na | 89.5% (17/19) | 0% (0/17) | na | 0% | na | 0% (0/19) | na | na |
|  | Marine Barged-Vacc. | Summer | na | na | na | 78.9% (15/19) | 40.0% (6/15) | na | na | na | 31.6% (6/19) |
|  | Marine Barged-Unvac. | Summer | na | na | na | 52.6% (10/19) | 50.0% (5/10) | na | na | na | 26.3% (5/19) |
|  | Marine – Vacc. | Summer | na | na | na | 57.9% (11/19) | 54.5% (6/11) | na | na | na | 31.6% (6/19) |
|  | Marine – Unvacc. | Summer | na | na | na | 36.8% (7/19) | 57.1% (4/7) | na | na | na | 21.1% (4/19) |
